# Distal end of Double-J ureteral stent position on ureteral stent-related symptoms: A systematic review and meta-analysis

**DOI:** 10.3389/fsurg.2022.990049

**Published:** 2022-08-12

**Authors:** Xingjun Bao, Fengze Sun, Huibao Yao, Di Wang, Hongquan Liu, Gonglin Tang, Xiaofeng Wang, Zhongbao Zhou, Jitao Wu, Yuanshan Cui

**Affiliations:** ^1^ Second Clinical Medical College, Binzhou Medical University, Yantai, China; ^2^ Department of Urology, The Affiliated Yantai Yuhuangding Hospital of Qingdao University, Yantai, China; ^3^ Department of Urology, Beijing TianTan Hospital, Capital Medical University, Beijing, China

**Keywords:** meta-analysis, ureteral stent, bladder midline, USRS, USSQ, IPSS

## Abstract

**Background:**

Most patients suffer from ureteral stent-related symptoms (USRS) caused by indwelling ureteral stents. Nevertheless, various medications to alleviate discomfort as well as novel stents are continually being developed, and in recent years, some researchers have believed that proper intravesical stent placement can relieve USRS.

**Objective:**

To determine appropriate intravesical ureteral stent position may alleviate USRS.

**Methods:**

Up to May 1, 2022, the PubMed, Embase, Scopus and Web of Science databases were thoroughly searched, and two independent reviewers included relevant studies that met the PICO (Patient, Intervention, Comparison, Outcome) criteria. Studies methodological quality were assessed by ROB2 and ROBINS-I. Ureteral stent symptom questionnaire (USSQ), international prostate symptom score (IPSS) and quality of life (QoL) was used to quantify the USRS. According to intravesical ureteral stent position, Group A was defined as the contralateral group, that is distal end of ureteral stent crossed the bladder midline, whereas Group B was classified as ipsilateral group, meaning stent end did not cross the midline.

**Results:**

Six studies incorporating a total of 590 patients were eligible. In terms of USSQ score, the meta-analysis showed that contralateral group was associated with a significant increase in USSQ total (MD, 17.55; 95% CI, 12.04 to 23.07; *P* < 0.001), urinary symptoms (MD, 2.74; 95% CI, 0.48 to 5.01; *P* = 0.02), general health (MD, 4.04; 95% CI, 2.66 to 5.42; *P* < 0.001), work performance (MD, 1.36; 95% CI, 0.75 to 1.98; *P* < 0.001) and additional problems (MD, 0.89; 95% CI, 0.47 to 1.32; *P* < 0.001) scores while not associated with a significant increase in body pain (MD, 3.13; 95% CI, −0.19 to 6.44; *P* = 0.06) and sexual matters (MD, 1.01; 95% CI, −0.03 to 2.06; *P* = 0.06). As for IPSS, although no significant differences in IPSS total (MD, 2.65; 95% CI, −0.24 to 5.54; *P* = 0.07) or voiding symptoms (MD, −0.84; 95% CI, −3.16 to 1.48; *P* = 0.48) scores were found, ipsilateral group was associated with a significant decrease in storage symptoms (MD, 1.92; 95% CI, 0.91 to 2.93; *P* = 0.0002). Furthermore, ipsilateral group was linked to a significant decrease in QoL score (MD, 1.00; 95% CI, 0.18 to 1.82; *P* = 0.02).

**Conclusion:**

This meta-analysis proven that correct intravesical stent position was critical, and patients with stents crossing the midline experienced more severe USRS than those who did not. Further high-quality randomized controlled trials are needed to corroborate our findings.

## Introduction

Double-J ureteral stent (DJUS), also known as double-pigtail stent, is now the most often utilized stent type in urology. With the advantages of its security and convenience, DJUS was extensively employed in the adjuvant treatment of urolithiasis, the release of upper urinary tract obstruction caused by various reasons and the expansion treatment of ureteral stenosis (1). The history of DJUS may be traced back to 1978, when Finney first revealed its benefits and application experience (2). Notwithstanding, because its material was not absorbable, an indwelling stent will ultimately induce urinary discomfort and even complications. It was no exaggeration to say that over 80% of patients with ureteral stents suffered one or more urinary tract symptoms, especially storage symptoms, urinary incontinence, dysuria and hematuria (3).

Causes and mechanisms of ureteral stent-related symptoms (USRS) remain unclear. The current studies supported the conclusion that various parameters, including stent design (4, 5), material (6), diameter (7), length and position (8, 9), may be related to the USRS. Moreover, some researchers thought that mechanical stimulation and retrograde pressure transmission from a stent were the core causes of USRS (10, 11). Several studies have focused on the relationship between stent position and USRS have appeared in recent years. Most researchers concurred that if the distal end of DJUS crossed the bladder midline, individuals would experience more severe USRS than those who did not (8, 12, 13). Furthermore, Lee and colleagues suspected that the intravesical appropriate stent placement was much more effective than drugs treatment for alleviating the USRS (14). In contrast, Abt et al. demonstrated stent position did not significantly influence the USRS (15). Some meta-analyses focusing on stent diameter and length have reported up to now, but there was still a void for stent position (16, 17).

Since its inception in 2003, the ureteral stent symptom questionnaire (USSQ) has been regarded as a sensitive and comprehensive tool for assessing USRS (18). Despite its lack of specificity, the International Prostate Symptom Score (IPSS) was commonly utilized in this evaluation (19). In our meta-analysis, we first used USSQ, IPSS and quality of life (QoL) scores to assess whether the distal end of DJUS crossing the bladder midline resulted in more severe USRS than those not crossing.

## Methods

We performed this systematic review and meta-analysis in accordance with the latest Preferred Reporting Items for Systematic Reviews of Interventions (PRISMA 2020) statements. **Supplementary file** provided the completed PRISMA 2020 checklist.

### Search strategy

**Figure 1:**
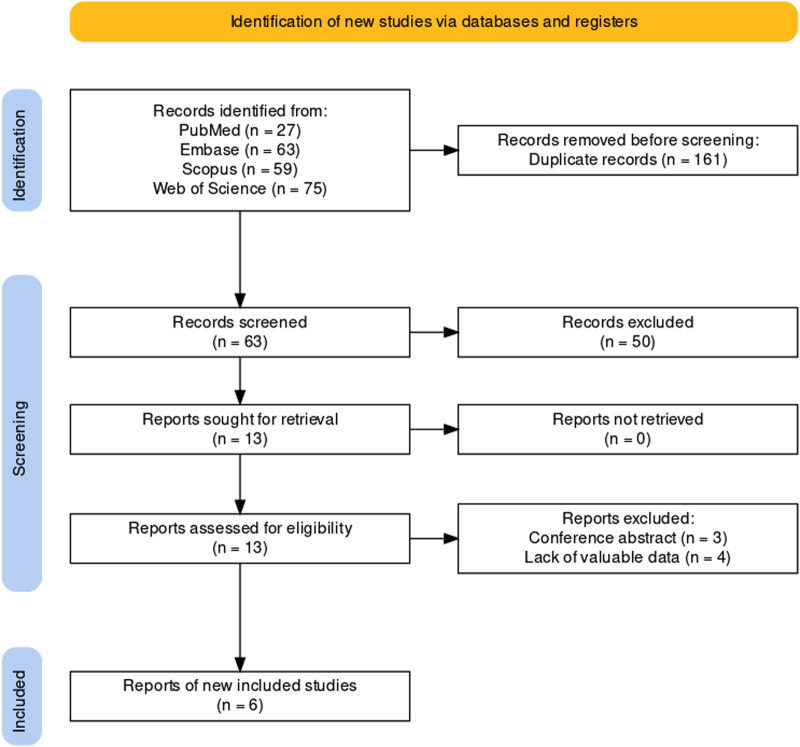
Flowchart of the literature search.

In PubMed, Embase, Scopus and Web of Science databases, search terms (“ureteral stent” AND “midline”) OR (“stent position” AND “symptoms”) were retrieved, and all literatures acquired up to 1 May 2022 were systematically reviewed. Case reports, editorials, conference abstracts, and non-English literature were all barred from consideration. Relevant articles from the selected articles' reference lists were also searched and reviewed. Two authors included relevant studies based on the PICO (Patient, Intervention, Comparison, Outcome) criteria. Any disagreements were resolved by discussion with a third author. The PRISMA flowchart is shown in Figure 1.

### Inclusion criteria

The study selection followed the PICO model (Patients: individuals with ureteral stents; Intervention: the distal end of ureteral stent crossed the midline of bladder; Comparison: the distal end of ureteral stent did not cross the midline of bladder; Outcomes: USSQ, IPSS and QoL). Furthermore, all included patients completed questionnaires and performed a plain radiograph of the kidney-ureter-urinary bladder prior to the stent removal procedure. The bladder midline was defined as a vertical line through the midline gap of the pubic symphysis based on imaging. Group A was defined as the contralateral group, that is distal end of ureteral stent crossed the bladder midline, whereas Group B was classified as ipsilateral group, meaning stent end did not cross the midline.

### Quality assessment

We used the revised Cochrane risk of bias tool for randomized trials (ROB2) and the risk of bias tool for non-randomized studies of interventions (ROBINS-I) to assess the risk of bias in randomized controlled trials (RCTs) and non-RCTs, respectively (20, 21). Disagreements among reviewers were resolved by consensus.

### Data extraction

The following data was gathered from the included studies: (1) First author's name and year of publication; (2) country of study; (3) sample size in each study group; (4) type, diameter, length and retention period of the stent; (5) primary outcomes, including USSQ, IPSS and QoL; and (6) age of the included population and indications of stent indwelling. Two authors worked independently to finish the procedure.

### Statistical analysis and meta-analysis

Outcomes analysis was performed with RevMan v.5.4.0 (Cochrane Collaboration, Oxford, UK). Using the Quantile Estimation (QE) method recently developed by McGrath et al. (22), the interquartile range was turned into mean and standard deviation (SD). The formula given by Zhang et al. was used to combine SD of different subgroups (23). The mean difference (MD) with 95% confidence interval (CI) was employed to describe continuous outcomes. The I-square (*I*^2^) and Q tests were used to assess heterogeneity among studies included. The random-effect model was utilized if the heterogeneity was considerable (*P* < 0.05 and *I*^2^ ≥ 50%). In contrast, fixed-effect model was selected for meta-analysis. For the overall effect, a *P* < 0.05 value was considered statistically significant.

## Results

### Characteristics of the individual studies

A total of 224 articles were retrieved. Following a review of the title and abstract, 214 papers were excluded. After further examination of the full-text, 4 articles (8, 9, 12, 24)were excluded due to the absence of available data. The remaining 6 papers (13–15, 25–27)were eventually included in the meta-analysis. Three of the included studies were randomized controlled trials (RCTs) (13, 14, 25), and three were prospective observational studies (15, 26, 27). The characteristics of the included studies are summarized in Table 1.

**Table 1:** Baseline characteristics of study and patient.

First author (publication year)	Country	Study type	Group (*n*)		Stent type	Diameter and length of Stent	Duration of stent (mean weeks)	Outcomes	Inclusion population
			A	B					
Taguchi (2022)	Japan	RCT	59	44	Inlay Optima stents (CR Bard Inc., USA)	6F, length adjusted by height	2	IPSS, QoL	Patients ≥20 years of age who underwent unilateral ureteroscopic lithotripsy were included
Mehra (2020)	India	prospective observational study	111	46	Not mentioned	5F, length adjusted by imaging examination	2	USSQ	patients between the ages of 18 and 70 years who underwent endoscopic ureteral lithotripsy were included
Inn (2019)	Malaysia	prospective observational study	22	24	Open tip ureteral stent (Allwin Medical Devices, USA)	6F, 24 cm	Not mentioned	USSQ	Patients ≥18 years of age who suffered stone obstruction or needed Post-intervention were included
Abdelaal (2016)	Egypt	RCT	51	127	Polyurethane JJ ureteric stent (Visiostar ureteric stent set, Germany)	Not mentioned	2.8	USSQ	patients ≥18 years of age who underwent extracorporeal shockwave lithotripsy, ureteroscopic lithotripsy, percutaneous nephrolithotripsy and endoscopic endopyelotomy were included
Abt (2015)	Switzerland	prospective observational study	40	13	Percuflex ureteral stents (Boston Scientific, USA)	6F, length adjusted by imaging examination and height	Not mentioned	USSQ	patients with a unilateral ureteral stent inserted for treatment of uretero- or nephrolithiasis were included
Lee (2010)	Korea	RCT	15	38	Percuflex ureteral stents (Boston Scientific, USA)	6F, length adjusted by height	1.4	IPSS, QoL	patients who underwent ureteroscopic ureterolithotomy for symptomatic ureteral calculi were included

Group A, crossing the bladder midline; Group B, not crossing the bladder midline; RCT, randomized controlled trial; IPSS, international prostate symptom score; QoL, quality of life; USSQ, ureteral stent symptom questionnaire.

### Risk of bias

**Figure 2:**
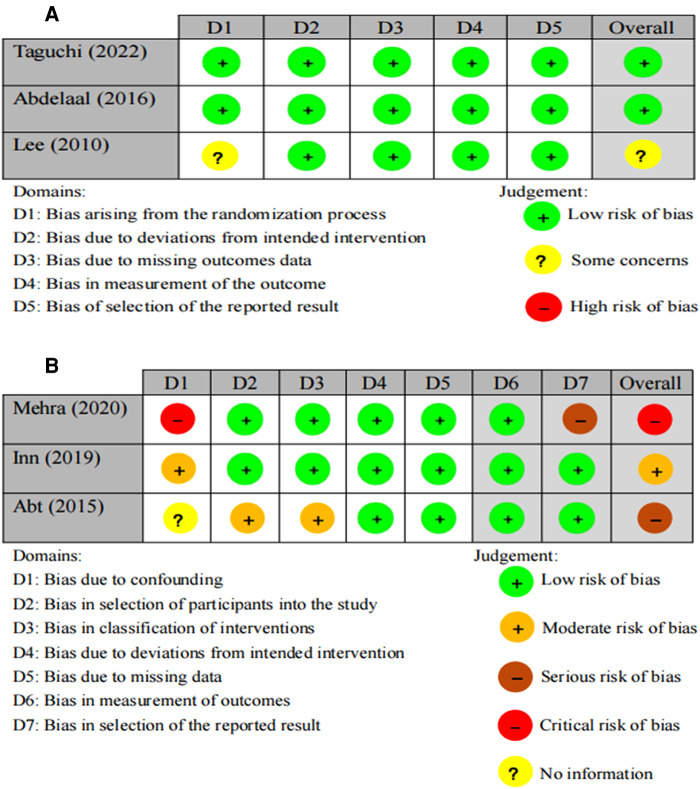
Risk of bias graph of the included studies. (**A**) Risk of bias rating of RCTs using ROB2. (**B**) Risk of bias rating of non-RCTs using ROBIN-I.

Figure 2 shows the detailed results of the risk of bias. Two RCTs (13, 25) explained their randomization protocol, and one study (13) performed intention-to-treat (ITT) analysis. According to RoB2, two (13, 25) of the three RCTs were classified as having a low risk of bias and one (14) as having some concerns due to an uncertain randomization sequence. Based on RoBIN-I, one non-RCT (27) had a critical risk of bias due to strong confounding variables and reporting bias. The remaining two non-RCTs (15, 26) were classified as having a serious risk of bias and moderate risk of bias, respectively.

### Primary outcomes of the individual studies

According to plain radiograph of the kidney-ureter-urinary bladder, patients with indwelling stents crossing the bladder midline were defined as group A, whereas patients with indwelling stents not crossing the midline were classified as group B.

#### Ureteral stent symptom questionnaire (USSQ)

**Figure 3:**
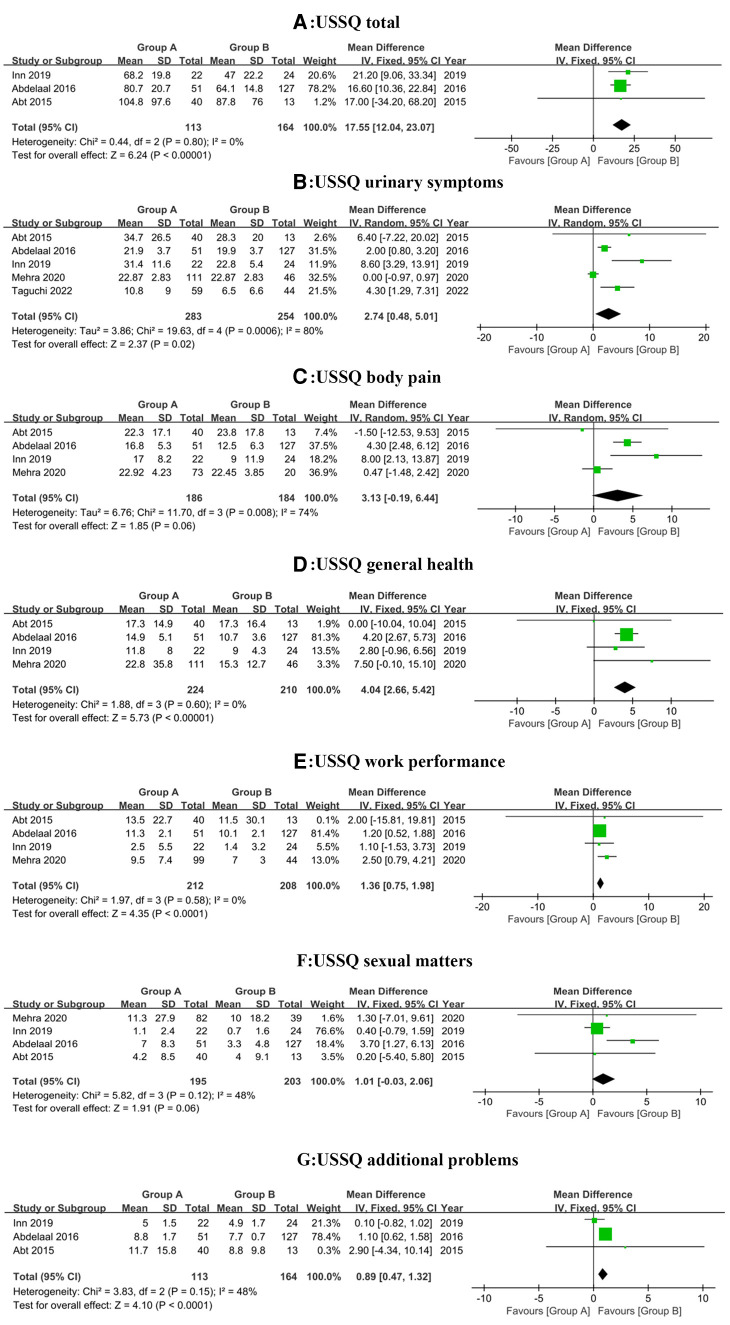
Forest plot depicting changes in USSQ total score and subscores.

Three studies (15, 25, 26), incorporating a total of 277 patients (113 in group A and 164 in group B), revealed the differences in USSQ total and additional problems score. There was no heterogeneity (*P* = 0.80, *I*^2^ = 0%) and low heterogeneity (*P* = 0.15, *I*^2 ^= 48%) among studies, hence the fixed-effect model was used for both analyses. The MDs was 17.55 (95% CI, 12.04 to 23.07; *P* < 0.001) and 0.89 (95% CI, 0.47 to 1.32; *P* < 0.001), respectively, as seen in Figures. 3A,G. These results demonstrated patients in group A experienced more severe discomfort than group B.

Five studies (13, 15, 25–27), incorporating a total of 537 patients (283 in group A and 254 in group B), revealed the difference in urinary symptoms scores. Because of the considerable heterogeneity (*P* = 0.0006, *I*^2 ^= 80%) among studies, the random-effect model was used. Compared with Group B, Group A was significantly associated with a higher score (MD, 2.74; 95% CI, 0.48 to 5.01; *P* = 0.02), as seen in Figure 3B. We came to the conclusion that patients with indwelling stents that did not cross the midline had better urinary symptoms.

Four studies (15, 25–27) disclosed changes in the score of the other four USSQ subgroups. The random-effect model was only employed to body pain score owing to the significant heterogeneity among studies (*P* = 0.008, *I*^2 ^= 74%), yet no significant difference (MD, 3.13; 95% CI, −0.19 to 6.44; *P* = 0.06) was found between the Group A (186 patients) and Group B (184 patients), as seen in Figure 3C. Furthermore, there were significant differences in general health (MD, 4.04; 95% CI, 2.66 to 5.42; *I*^2 ^= 0%; *P* < 0.001) and work performance (MD, 1.36; 95% CI, 0.75 to 1.98; *I*^2 ^= 0%; *P* < 0.001) scores while no significant difference in sexual matters (MD, 1.01; 95% CI, −0.03 to 2.06; *I*^2 ^= 48%; *P* = 0.06) score. No heterogeneity was found among studies and the fixed-effect models were selected in the three subgroups, which included a total of 434 (224 in group A and 210 in group B), 420 (212 in group A and 208 in group B) and 398 patients (195 in group A and 203 in group B) separately, as seen in Figures 3D–F. All in all, except for body pain and sexual matters, patients with indwelling stents not crossing the midline reported greater satisfaction in general health and work performance than patients with indwelling stents crossing the midline.

#### International prostate symptom score (IPSS)

**Figure 4:**
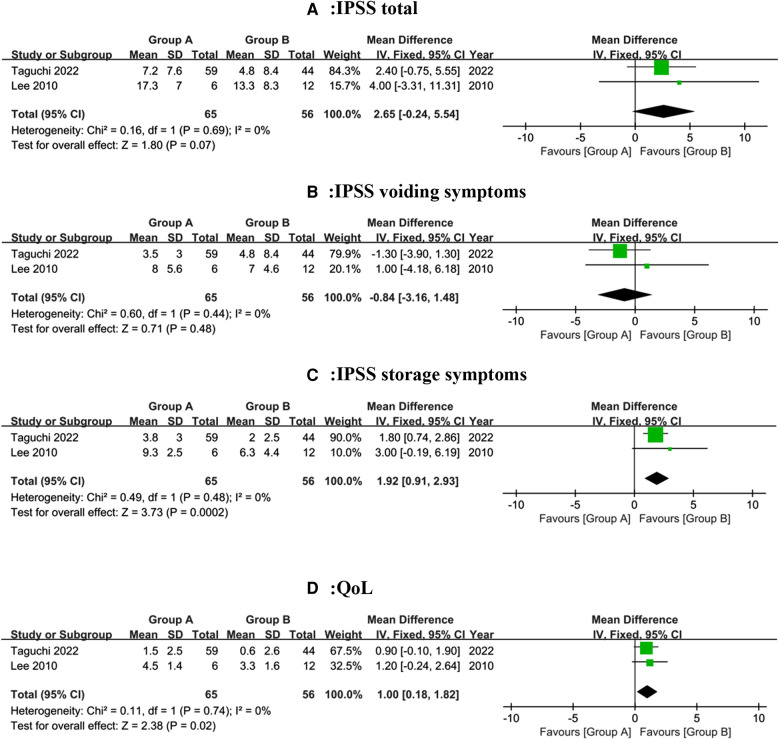
Forest plot depicting changes in IPSS and QoL scores.

Two studies (13, 14), incorporating a total of 121 patients (65 in group A and 56 in group B), revealed the changes in IPSS total and it subgroups score. No heterogeneity (*I*^2^ = 0%) was found among studies, and the fixed-effect models were applied to meta-analysis. IPSS total and voiding symptoms scores by a mean of 2.65 (95% CI, −0.24 to 5.54; *P* = 0.07) and −0.84 (95% CI, −3.16 to 1.48; *P* = 0.48) respectively were no significant differences, as seen in Figure 4A,B. Intriguingly, the MD of storage symptoms subscore was 1.92 (95% CI, 0.91 to 2.93; *P* = 0.0002), as shown in Figure 4C. Although IPSS total score and subscore of group A were higher than group B, we only had evidence to conclude that individuals in group A experienced more severe storage symptoms.

#### Quality of life (QoL)

Two studies (13, 14), incorporating a total of 121 patients (65 in group A and 56 in group B). Revealed the difference in QoL score. There was no heterogeneity (*P* = 0.74, *I*^2 ^= 0%) among studies, so the fixed-effect model was used for the meta-analysis. The results of integrative data analysis revealed that patients in Group B were associated with a significant decrease in QoL score (MD, 1.00; 95% CI, 0.18 to 1.82; *P* = 0.02; Figure 4D). Therefore, patients in group B had a greater quality of life than group A.

## Discussion

Despite the wide range of indications for DJUS, the ensuing USRS were indeed vexing (1). Hao et al. showed that approximately 19.6% of individuals with ureteral stents experienced one or more discomforts, whereas Joshi and colleagues reported that up to 80% of patients with ureteral stents suffered a variety of urinary symptoms, with storage symptoms, incontinence, dysuria and hematuria being the most bothersome (3, 28). However, the pathogenesis of USRS has not been fully elucidated to date. It has been suggested that stent-related flank pain was due to the backflow of urine from the stent into the renal collecting system during urination. In addition, stent-related irrigative symptoms may be attributed to irritation of the mucosa of the bladder associated with stent migration due to active during the day (1, 10). In a word, the mechanisms of the USRS were still poorly studied, and treatment options for USRS were limited.

Although pharmacologic interventions were the mainstay of treatment for USRS, it adverse effects caused some patients to fail to take their prescription (29). At present, experts studies have revealed that stent material, shape, diameter, length, and position all had the potential to influence the USRS (13). RANE et al. (24) proposed in 2001 that stent position was linked with the USRS. This study included 60 patients showed that the incidence of urinary urgency and asymptomatic cases was as high as 72% and 33.3% respectively in the contralateral group compared to 33.3% and 66.6%, respectively, in the ipsilateral group, and the differences were statistically significant. Furthermore, AL-KANDARI et al. research (8), which included 120 individuals, reported that 53 individuals (88%) in the contralateral group had moderate to severe dysuria compared to 11 individuals (18%) in the ipsilateral group (*P* < 0.001). It was noteworthy that correct intravesical stent placement has been repeatedly proven to improve the USRS (13, 26, 27). Thus we included 6 studies with 590 individuals to explore the impact of stent position on the USRS using a meta-analysis of USSQ, IPSS, and QoL score. This is, to the best of our knowledge, the first literature review and meta-analysis evaluating the effect of stent position on the USRS. The analyses demonstrated that contralateral group had higher USSQ, storage symptoms, and QoL scores than ipsilateral group. This also served as a reminder to urologists to carefully inspect indwelling stents to ensure that they were in the proper location.

Besides, a retrospective study (12) found that the contralateral group had worse overactive bladder symptom score (OABSS) total score and sub scores than the ipsilateral group, and multivariate analysis revealed that stent position was an independent predictor of the USRS. Remarkably, Lee et al. (14) demonstrated that correct stent position was more significant than medication treatment for relieving the USRS in a prospective randomized study. This begs the question, what exactly causes patients with stent crossing the midline of the bladder to have more severe USRS?

Distal end of ureteral stent crossing the midline was associated with more severe USRS, possibly as a result of direct physical contact with the intravesical stent with the contralateral bladder wall (30). Our study demonstrated the contralateral group had more severe storage symptoms, which were directly related to irritation of the bladder trigone. It was not difficult to imagine that intravesical stent crossing the midline would increase the risk of irritation to the bladder trigone, especially when the patient was active (11). Interestingly, there was no significant link between ureteral stent length and intravesical stent position. This might be due to the fact that intravesical stent location varies with time and patient position, and a study has indicated that shifts from ipsilateral through midline to contralateral were more prevalent, which could also explain why around 80% of patients suffered USRS (31). A study (17) found that there was no significant correlation between stents with small diameter and stent migration, therefore whether using stents with smaller diameters and without crossing the bladder midline may effectively relieve USRS has to be examined further.

In recent years, drug-eluting expandable metal stents and biodegradable stents have emerged owing to the prevalence of USRS, stent encrustation, stent migration and stent-related urinary tract infection (32, 33). To promote stent development and avoid ureteral stent migration to the contralateral side to trigger severe USRS, can we focus stent innovation on limiting stent migration? All in all, stent related technology is constantly improving, and we will be able to totally eradicate stent related symptoms.

Our analysis had apparently limits. We were unable to incorporate more high-quality RCTs to support our findings due to a paucity of previous research. The studies included in the meta-analysis may have biases. The patient characteristics, stent parameters, stent duration, and questionnaire scoring time was not consistent. These variables may have an impact on the primary outcome of our study. But, to our knowledge, this was the first systematic review and meta-analysis assessing the effect of stent position on the USRS.

## Conclusion

In conclusion, our meta-analysis revealed that patients with stents crossing the midline suffered more severe discomforts in subgroups such as urinary symptoms, general health, work performance, additional problems, storage symptoms, and QoL. When indwelling a ureteral stent, urologists must take the time to ensure that the stent is properly positioned. However, better quality randomized controlled trials are urgently required to validate our outcomes.

## Data Availability

The original contributions presented in the study are included in the article/**Suplementary Material**, further inquiries can be directed to the corresponding author/s.
